# Potential Fluid Biomarkers for the Diagnosis of Mild Cognitive Impairment

**DOI:** 10.3390/ijms20174149

**Published:** 2019-08-25

**Authors:** Vo Van Giau, Eva Bagyinszky, Seong Soo A. An

**Affiliations:** Department of BionanoTechnology, Gachon University, 1342 Sungnam Daero, Sungnamsi 13120, Korea

**Keywords:** mild cognitive impairment, Alzheimer’s disease, biomarkers, diagnosis

## Abstract

Mild cognitive impairment (MCI) is characterized by a level of cognitive impairment that is lower than normal for a person’s age, but a higher function than that that observed in a demented person. MCI represents a transitional state between normal aging and dementia disorders, especially Alzheimer’s disease (AD). Much effort has been made towards determining the prognosis of a person with MCI who will convert to AD. It is now clear that cerebrospinal fluid (CSF) levels of Aβ40, Aβ42, total tau and phosphorylated tau are useful for predicting the risk of progression from MCI to AD. This review highlights the advantages of the current blood-based biomarkers in MCI, and discusses some of these challenges, with an emphasis on recent studies to provide an overview of the current state of MCI.

## 1. Introduction

**Figure 1:**
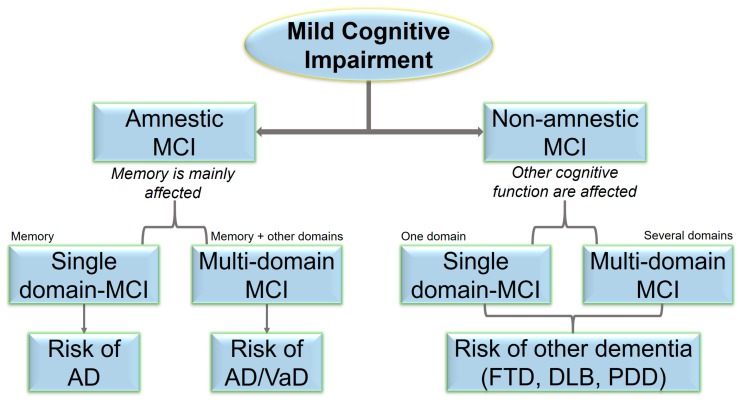
Subtypes of MCI and their risk for neurodegenerative diseases. MCI, mild cognitive impairment; AD, Alzheimer’s disease; VaD, vascular dementia; FTD, frontotemporal dementia; DLB, dementia with Lewy bodies; PDD, dementia in Parkinson’s disease.

Memory and cognitive impairments are common among elderly patients, and are possible risk factors for dementia. Mild cognitive impairment (MCI) became a novel topic in current research with the hypothesis it represents “grey line” or “transitional zone” between normal cognition and dementia, such as Alzheimer’s disease (AD) [1,2]. Approximately 10–15% of individuals with MCI develop dementia every year, compared with 1–2% in unaffected individuals [3,4]. The criteria for MCI was established in 1999 at the Mayo Alzheimer’s Disease Center. An MCI diagnosis can be made if patients meet several criteria. First, they should exhibit a low degree of memory impairment, or abnormal memory for their age. However, their general cognitive function should be relatively normal, and they should be able to perform their daily activities. Individuals with MCI do not meet the diagnostic criteria for dementia [5]. MCI is a heterogeneous disease, and it can be variable in terms of gender, age, and subtype. Two types of MCI can be distinguished: amnestic- and non-amnestic MCI (Figure 1). In the amnestic form, memory impairment is the most prominent feature. In the non-amnestic form, memory may remain normal, but other cognitive dysfunctions may be observed, such as dysfunctional attention, language, or executive functions [6]. Individuals with amnestic MCI may have a higher risk of developing AD, while non-amnestic MCI patients may have a higher chance of developing other types of dementia, such as frontotemporal dementia or dementia with Lewy bodies (DLB). Other classifications include single- or multiple-domain MCI, in terms of whether one or more cognitive domains are affected, respectively. Multiple-domain MCI may be a high risk factor for progression into dementia than single-domain MCI. Multi-domain amnestic MCI can also be an AD risk, but vascular dementia or AD with vascular dysfunctions can also occur. Non-amnestic MCI patients may have a higher risk of developing other types of dementia, such as frontotemporal dementia, dementia with Lewy bodies (DLB), or Parkinson’s dementia (PDD) [7,8,9].

MCI diagnosis presents several controversies, since the prevalence and incidence of MCI is variable. One issue is the uncertainty regarding whether patients with mild depression should be considered as having MCI, since depression may be risk factor for memory impairment. Another important challenge is the separation of dementia and MCI, since the “impairment in functional ability” may be difficult to define [10]. In addition, MCI does not always progress to AD, and in some individuals it might persist without ever transforming into AD. In some cases, MCI seems to be reversible [11].

**Figure 2:**
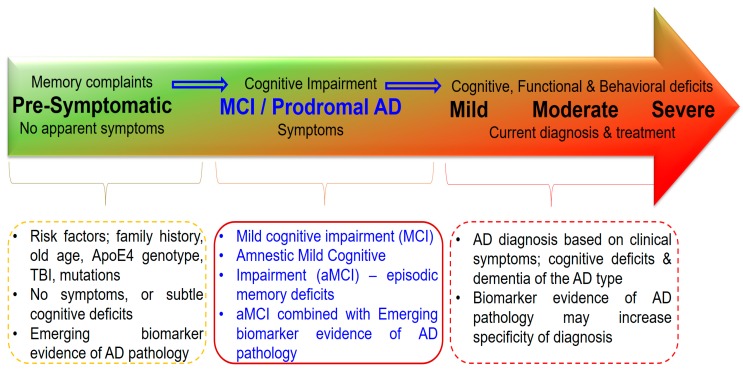
Progression from normal aging to Alzheimer’s disease or another dementia.

Diagnosis of MCI is important, because it could be a pre-dementia phase. MCI could be considered as a risk factor for AD, but also a prodromal phase of AD (Figure 2). It is essential to define the criteria for patient identification accurately, specifically those who have risk of disease progression, whose condition will remain “stable”, and whose cognitive function will reverse into a normal state [2]. Since the majority of treatment strategies are more effective in the presymptomatic stage of dementia, more studies have been performed on the diagnostic strategies for MCI. Using the genetic, proteomic and imaging markers could be essential for disease risk prediction [12]. In the preclinical stage of dementia, degree of cognitive decline is unremarkable, and individuals may still present normal cognitive abilities. Among the elderly, preclinical dementia often remains undiagnosed, even though it can affect normal aging [13]. Cognitive deficits may be detectable years or even decades prior to the clinical symptoms of dementia. Changes in the levels of biomarkers in body fluids and in specific brain regions may allow the detection of these cognitive alterations even before the appearance of MCI. Different proteomic and genetic markers may result in more accurate prediction regarding who will develop dementia, e.g., AD, in the future [14]. This review summarizes the candidate biomarkers that may be useful in MCI diagnosis. In addition, the diagnostic methods and challenges in diagnosing will also be discussed.

## 2. Diagnostic Tools of MCI and Preclinical Dementia

Several research approaches have been undertaken to diagnose MC using neuropsychiatry, imaging or different proteomic and genetic markers. MCI diagnostic research falls into two categories: (1) cross-sectional research involving screening for markers that can distinguish MCI from normal controls and (2) longitudinal studies that screen MCI patients for prediction of conversion to dementia [15]. Neuropsychological tests can be performed on patients to check their episodic memory, such as verbal and visual memory, and associative learning. Semantic memory can also be tested by screening for their fluency and general intelligence [16]. Imaging techniques have been widely used for the characterization of AD patients, but they could also be useful in monitoring changes in the brain. Brain imaging could provide more information about risk factors that could be used in clinical and preclinical diagnosis. In addition, imaging approaches could measure brain alterations and possible disease progression even in the absence of noticeable cognitive impairment [17,18]. Structural-functional magnetic resonance imaging (MRI), positron emission tomography (PET) and single-photon emission computed tomography (SPECT) screening may be used in individuals with preclinical stage of dementia or with MCI [15]. Amyloid aggregates and neurofibrillary tangles in AD could be associated with neuronal death and cerebral atrophy in specific brain areas. In MCI and AD, the hippocampus and entorhinal cortex may be the first regions affected by atrophy. Volumetric MRI could be useful in structural imaging of AD patients. T1-weighted MRI imaging could be useful for examining the topographic distribution of atrophy in the cortex. T2-weighted MRI provides a quantitative analysis of atrophy, but may not be useful in the differential diagnosis of MCI and AD. Functional MRIs could be used for more advanced applications for detecting disease-specific alterations in brain functions. Using structural and functional MRI could predict AD onset in individuals with MCI [19]. Amyloid PET could be useful for detecting the amyloid deposits in MCI patients and for monitoring the amyloid burden in the brain [20]. In addition to technologies, biomarkers of body fluids should also be used for accurate diagnosis of cognitive impairment. Establishing biomarkers in the blood as well as the cerebrospinal fluid (CSF) biomarkers could be critical not only for disease diagnosis, but also for prediction of possible dementia progression. A combination of different known disease markers (such as decreased amyloid, elevated Tau levels, or elevated Tau/amyloid ratio) in CSF may have a potential clinical utility as biomarkers of the disease.

## 3. Multiple Approaches to Blood-Based Markers

MCI seems to be an etiologically heterogeneous syndrome characterized by memory performance below the age norm, otherwise unimpaired intellectual functioning, and well-preserved activities of daily living. Several MCI individuals may progress to AD, but they can also progress to other type of dementia. In addition, in many MCI individuals, cognitive dysfunctions may revert back to normal. The prevalence of MCI among the population of > 65 years in age is about 10–25% in industrialized countries [21]. Elderly individuals with MCI constitute a high-risk population of developing dementia, especially AD. The annual conversion rate of MCI to AD has been estimated in general to be about 10–15% [22]. When considering all forms of MCI, the incidence rates of 51 and 76.8 per 1000 person-years have been reported [21]. The most noteworthy risk factors of incident MCI are higher age [23,24], lower education [25] and hypertension [26]. Research with a focus on specific biomarkers that predict incipient MCI is crucial. It is also important to examine whether blood-based biomarkers are more useful than imaging data for detecting an increased amyloid burden in MCI.

## 4. Diagnostic Approaches and Tools of MCI

Several biomarkers have been described for the diagnosis of MCI that could predict the possible development of dementia. Biomarkers could be categorized through a binary scheme known as the A/T/N system. This classification was proposed by National Institute on Aging Alzheimer’s Association (NIA-AA), and can also be used for dementia prediction on a single individual. Class “A” corresponds to the Aβ marker, class “T” corresponds to the Tau marker, and class “N” stands for neurodegeneration. Each category can be rated as positive or negative. The A/T/N system was organized through measurement of biomarkers in plasma and CSF, as well as through imaging analyses. This A/T/N system could be useful for categorizing the multi-domain biomarkers, and predicting possible AD progression [27,28]. Ekman et al. revealed that the levels of amyloid- tangle pathology and neurodegeneration increased in individuals with progressive MCI and AD patients compared with those with stable MCI and unaffected control individuals. A−T−N− pattern was higher in healthy individuals and also in individuals with stable MCI (Table 1). Overall, amyloid positive (A+) individuals showed a staging pattern where the A+/T+/N+ pattern was most common, followed by the A+/T+/N− pattern, and finally the A+/T−/N− pattern was less common [27]. In addition, the A/T/N classification showed a stepwise increase in numbers of A+/T+/N+ profiles from HC (12%), via MCI-S (29%), to MCI-P (54%), and finally the highest number for AD (63%) [27].

Markers from biofluids can be analyzed using different assays. A multimer detection system (MDS) was initially developed to detect prion aggregates [29,30,31]. This method consists of a sandwich enzyme-linked immunosorbent assay (ELISA), which uses two unique epitope-overlapping antibodies that can detect oligomer forms of different protein aggregates [30], and can also be used in dementia diagnosis and prediction [32,33]. Screening amyloid oligomers by MDS in plasma may be useful in the diagnosis of MCI and its conversion to AD [33]. A magnetic droplet immunoassay was developed for oligomer detection, which could be a sensitive and simple method for detecting amyloid beta oligomers. This assay is based on MDS methods, and consists of a microfluidic device with a micro-pillar structure, and it could be an effective method for oligomer Aβ quantification [34]. Quanterix has developed a single molecule array called Simoa. This approach is based on a digital bead-based ELISA assay, but has the potential to be a more sensitive method [35]. Simoa assays has been successfully used for monitoring Tau [36], Aβ peptides [37] or neurofilament light chain (NFL) protein [35].

Despite great controversy in the literature, the systematic assessment of these biomarkers has been incorporated into recent revisions of AD and MCI diagnosis. In Taiwan, a hyper-sensitive assay, superconducting quantum interference device (SQUID) was developed, and may be able to detect the interaction between biomarkers and magnetic nanoparticles [38]. This method was able to measure the concentration of both Ab42 and Tau, not only in the CSF, but also in the plasma. This method may be useful for the diagnosis of AD and dementia, and may even recognize preclinical AD and MCI [39].

## 5. Mild Cognitive Impairment in Relation to CSF Biomarkers

There is great interest in finding diagnostic tools that could detect an increased risk of AD development in MCI subjects. Since the pathophysiological events leading to dementia precede the clinical symptoms, biomarkers for MCI have become an area of great interest for both researchers and clinicians alike [40]. Both structural and functional neuroimaging, as well as CSF Aβ1-42 and Tau, have shown promising results in improving the prediction of which MCI subjects will develop AD. Cerebrospinal fluid biomarkers, such as Aβ1-42, Tau and pTau, might be useful in identifying MCI subjects at risk of developing AD (Table 2) [41,42,43,44,45,46]. A combination of Aβ1–42 and Tau displayed sensitivity as high as 95% and specificity of 83% in detecting MCI subjects that developed AD [47]. However, [11C] PIB PET imaging might be more sensitive than CSF biomarkers in its ability to discriminate prodromal AD patients [48]. Biomarker measurements mainly consist of brain amyloidosis (amyloid positron emission tomography, cerebrospinal fluid Aβ42) and neurodegeneration (medial temporal atrophy in MRI, fluorodeoxyglucose positron emission tomography, CSF tau) [49]. In the early stages of AD, patients may present with mild but persistent (and often progressive) cognitive deficits, albeit not severe enough to warrant the diagnosis of dementia (e.g., patients with amnestic MCI) [50,51].

Besides amyloid peptides and Tau, additional markers were also examined in CSF, which could be used in A/T/N framework analysis. Fatty acid binding protein 3 (Fabp3), Neurofilament (NfL), and IL-10 have been suggested as potential candidates for diagnosis of AD. Reduced IL-10 in the CSF of MCI patients may accelerate cognitive decline. These markers may be useful in distinguishing individuals with MCI from those with AD or from those who are at a risk of MCI-AD conversion [52]. Trefoil factor 3 (TFF3), a substrate of NOTCH signaling, may also serve as a candidate CSF marker for cognitive decline. Low levels of TFF3 are associated with a higher degree of atrophy in the hippocampus and expansions to the ventricles in amyloid-positive individuals [53].

In summary, evidence from studies on the effect of pre-analytical handling on biomarkers of MCI suggest that use of the CSF Aβ1-42, t-tau and p-tau as potential biomarkers for MCI or AD-MCI CSF would improve the interpretation of CSF amyloid biomarker results, by reducing the impact of these factors on outcome. The use of the CSF Aβ1-42, t-tau and p-tau could therefore contribute toward pre-analytical standardization, allowing for the use of CSF MCI biomarkers in routine clinical practice. The main disadvantage of the use of those biomarkers is economical and not interpretational in nature. Considering the laboratory costs of the MCI biomarkers, the inclusion of Aβ40 increases the total costs of the diagnostic work-up and treatment of patients with suspected MCI assessed at specialized memory clinics. Furthermore, obtaining CSF from elderly individuals on repeated occasions is no easy task. The volume of CSF sample use to perform this additional test needs to be carefully considered. Table 3 introduces the benefits and disadvantages of diagnostic tools, used in diagnosis of mild cognitive impairment.

## 6. Other Potential Biological Fluids

Peripheral biomarkers for MCI diagnosis are also important. The use of tau levels in blood plasma as potential biomarker for cognitive impairment has failed due to the extremely low levels of tau. Amyloid-beta peptides could be used more effectively as predictive biomarkers. Aβ42 peptides may help to predict the changes in normal cognition or MCI to AD transition, but Aβ peptides themselves may not serve as effective biomarkers. However, the ratio of Aβ42/Aβ40 could be an effective marker for predicting the risk of MCI/AD development [54,55].

Clusterin has been verified as a risk factor for AD, and plasma clusterin levels may be altered during neurodegeneration [56]. Clusterin can play a significant role in neurodegeneration. Differences have been observed between the plasma concentration of patients with MCI and cognitively normal individuals. Higher levels of plasma clusterin may be related to lower levels of brain atrophy (temporal brain volume) in patients with MCI. In addition, MCI patients with higher clusterin levels may have higher risk for AD progression. It has been suggested that clusterin could be a prognostic marker for AD prediction [57,58]. This biomarker may provide clinicians and caregivers with information on disease severity and progression in patients with AD, and give them a clear picture of the future to assist with designing a suitable care plan for patients with MCI-AD.

Elevated lipid levels in plasma have been shown to play a role in several human diseases, and could also be related to cognitive decline. Initial studies failed to find an association between plasma lipids and MCI [59]. In 2012, Yin et al. revealed that triglycerides (TG) were negatively associated with MCI, and could preserve cognitive decline in the elderly. This study did not find any association between cholesterol and cognitive impairment [60]. In 2016, He et al. analyzed the total cholesterol (TC), high-density lipoprotein cholesterol (HDL-C), low-density lipoprotein cholesterol (LDL-C), and triglyceride (TG) levels in patients with MCI compared with healthy individuals. This study found higher TC levels in MCI individuals compared with controls; however, this finding may be contradictory. Previously, other studies revealed that high TC might have a protective effect against AD or cognitive impairment. This study observed reduced HDL-C in MCI patients, suggesting that HDL-C can prevent amyloid aggregation and cognitive impairment. A similar association was found between plasma TG levels and MCI, suggesting that TG may also have a protective effect [61]. The relationship between TGs and amyloid-β may be explained through role of TGs in the peripheral lipoprotein transport of Aβ. Further studies are required to investigate the biomarker.

Misfolded p53 has been suggested to be a strong risk factor for MCI to AD progression. Elevated levels of unfolded p53 were found in both AD and MCI individuals, compared with that in normal controls. High unfolded p53 levels in the blood could be a predictive marker from MCI to AD conversion, even in the presymptomatic stage [62].

Immune mechanisms may play a significant role in AD onset, and inflammatory biomarkers may be useful in disease diagnosis [63]. Inflammatory molecules in plasma may be associated with early stages of cognitive decline, but there have been conflicting findings regarding their association [64,65]. Plasma lactate is associated with systemic inflammation, and may be an indicator for mitochondrial dysfunctions. Lactate may be a possible pro-inflammatory molecule, and may increase the production of reactive oxygen species (ROS). In a Chinese population, higher lactate levels in plasma may result in higher MCI prevalence [66]. A recent study found an association between MCI and inflammatory markers in plasma, including tumor necrosis factor-α (TNFα), vascular endothelial growth factors A (VEGF-A), C-peptide and plasminogen activator inhibitor (PAI-1). TNF-α is a pro-inflammatory molecule, and its elevated levels in MCI patients may reflect neuronal dysfunctions/loss. Expression of TNF-α may be induced by amyloid peptide, and its expression may be increased during disease progression. Levels of PAI-1, C-peptide and VEGF-A may be associated with vascular and metabolic dysfunction [67]. Several interleukins, such as IL-10, IL1-beta, IL2 and IL4 were found to be elevated in the plasma of MCI patients relative to that in unaffected individuals. These findings suggested that inflammation might occur in the early stage of neurodegeneration. Interleukins may help to predict what kind of neurodegenerative disease could occur in patients with MCI [68]. Higher levels of IL6 and IL10 in the blood are associated with reduced cognitive functions, however their role in the diagnosis of MCI is questioned [69]. Antibodies may also play a role in cognitive dysfunctions. By penetrating to the brain through the blood brain barrier, they could result in either reversible or irreversible brain damage. Antibody-associated irreversible brain damage may continue when antibodies are not present in the brain. Antibodies were verified to play a role in autoimmune brain diseases, such as systemic lupus erythematosus. However, these antibodies may also disturb cognitive functions [70]. N-Methyl-d-aspartate glutamate receptor (NMDA-R) antibodies may be involved in different types of neurodegenerative diseases, such as AD or vascular dementia. NMDA-R antibodies could disturb the blood-CSF-barrier, and promote the onset of cognitive decline [71]. Autoantibodies were suggested to be potential markers, which could distinguish MCI individuals from those who developed early AD. Autoantibodies are present in human sera, and have been suggested to be useful marker in the differential diagnosis of neurodegenerative diseases [72].

Autoantibodies might have dual role in AD, they either exert a protective effect against AD pathology by inducing tissue damage through autoimmune reactions or enhance neuroprotection by inhibiting toxic aggregation and promoting amyloid clearance [73]. A majority of studies revealed that Aβ autoantibodies could be present either in an unbound form or as a part of antigen-antibody complexes. Several studies have revealed reduced levels of unbound Aβ autoantibodies [74,75,76], which may reflect the immune dysfunctions, such as T helper cell impairment [75]. Autoantibodies may play a role in clearance of amyloid aggregates, and dysfunction in their mechanisms may occur in case of AD. In serum, the levels of autoantibodies against Aβ1–15 were reduced in AD patients, compared with the unaffected controls. Aβ1–15 antibodies were correlated with the ε4/ε4 genotype, as their levels were reduced in both controls and AD patients containing the homozygous ε4 allele [77]. Other studies revealed elevated Ab autoantibodies in case of the cognitive dysfunctions. Storace et al. reported that autoantibodies for Aβ1–42 were significantly higher in plasma of amnestic MCI individuals and MCI-AD patients, compared with the unaffected individuals. Thus, antibodies be used as possible predictive marker for AD progression [78]. Autoantibodies against Tau have not been extensively studied among patients with neurodegenerative diseases. It may be possible that levels of anti-phosphorylated-tau autoantibodies (IgM) could be higher in the case of neurodegeneration. Their increased levels may be associated with neuroinflammatory process, but it remains unclear, whether this increase is pathogenic or protective [79,80].

In addition, synaptic pathology occurs early in AD development suggesting that alterations in the axonal or synaptic compartment are a primary event in the progression of the disease [81,82]. Currently, less well-studied but promising “emerging” CSF biomarkers of other disease processes have been reported [83], which are altered in individuals with symptomatic AD relative to controls, including neurogranin (Ng) [84,85,86], chitinase-3-like protein 1, also known as YKL-40 [87,88], synaptosomal-associated protein-25 (SNAP-25) [89], and visinin-like protein 1 (VILIP-1) [90,91]. Neurogranin is the dendritic analog of presynaptic neuromodulin in the postsynaptic membrane [92]. Changes in neurogranin levels have been reported to occur in the brain [93] and CSF [94]. Recently, a trend towards increasing levels of neurogranin, T-tau and P-tau181 was observed in CSF from MCI patients relative to controls [82], which might reflect synaptic degeneration. In addition, studies have suggested that increased CSF neurogranin levels might even be predictive of progression from MCI to AD; thus, this protein has also been discussed as a potential AD biomarker [84,85]. Hence, CSF neurogranin in combination with the established AD biomarkers could be a valuable tool for the early diagnosis of AD and for identification of incipient AD in patients with MCI. There are only a few studies related to concentrations of YKL-40 in the CSF of patients with full symptomatic AD and predementia stages as well as in other types of dementia [88,95,96]. Increased concentrations of YKL-40 were observed not only in fully developed AD, but also in the early stages of AD. The increase in YKL-40 concentrations in CSF were in very mild in mild dementia subjects compared with the cognitively normal individuals. A similar finding was this observed in in patients with preclinical AD [87]. Increased levels of YKL-40 predicted progression from MCI to symptomatic AD and other types of dementia as measured by annual assessment of MMSE within follow-up [87]. This finding suggests diagnostic usefulness of CSF levels of YKL-40 in AD and for the distinction between the stable phase of MCI and patients who progressed to vascular dementia and AD [97,98]. Taken together, early abnormalities in CSF tTau, pTau, SNAP-25, VILIP-1, and YKL-40 that may be useful biomarkers of AD brain pathology in its early stages and predict dementia onset including MCI. However, these biomarkers for MCI and AD still require additional studies to improve the diagnostic accuracy of CSF analysis further.

## 7. Micro RNA (miRNA) in the Diagnosis of MCI and the Prediction of AD

Micro RNAs (miRNA) are short (19–24 nucleotide long) non-coding RNAs that regulate gene expression. MiRNAs have been shown to play a significant role in brain and neuronal development. Brain aging is associated with altered miRNA expression. For example, miRNAs can modulate synaptic plasticity, inflammatory processes or lipid metabolism. During aging, the risk of cognitive decline and neurodegenerative diseases increase. Altered expression of miRNAs may predict the onset of cognitive dysfunctions [99]. Detecting miRNA in the plasma could be a promising approach for the early detection of MCI. Cell-free circulating miRNAs may be present in enriched in brain, and could reflect the early neurodegenerative changes, and may predict the MCI/AD onset during the pre-symptomatic stage [100].

**Figure 3:**
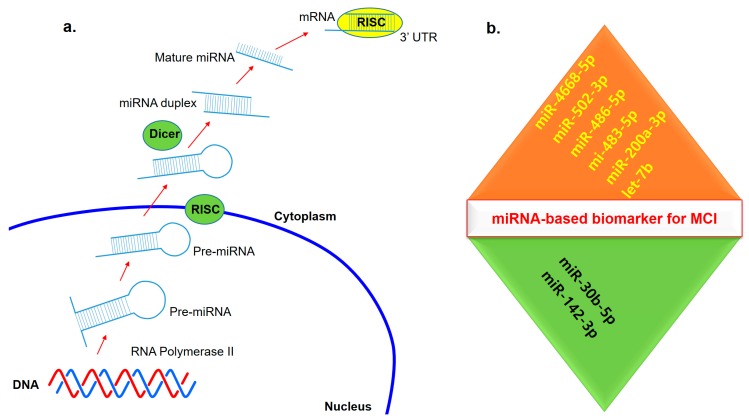
Non-coding RNAs in mild cognitive impairment (MCI). (**a**) The mechanisms of miRNA-mediated gene regulation. (**b**) Some common miRNA that are downregulated (green triangle) and upregulated (orange triangle) in blood serum, blood plasma, and cerebrospinal fluid (CSF) of MCI patients compared with normal controls.

Recently, the role of miRNAs in the early diagnosis for neurodegenerative disorders has been extensively studied. MiRNAs (Figure 3) may be stable disease markers, present in different body fluids, such as plasma, serum, urine, and CSF [101,102]. The miR-132 and miR-134 families have been suggested as possible predictive markers for the onset of MCI in preclinical stage. They can also reflect age-related changes in the brain, but might not be able to distinguish the patients with MCI from those with AD [103]. A combination of four miRNAs (miR-132, miR134, miR-491-5p and miR-370) have been identified as strong markers for MCI detection. This study achieved high accuracy for differentiating MCI individuals from controls. These miRNA pairs may be useful in detection of early AD stages [100]. Studies on serum miRNAs found that levels of miR-93 and miR-146a were elevated in MCI individuals, while those of miR-143 levels were reduced. All these markers were downregulated in AD patients, suggesting that these miRNAs may be important in the initiation of AD-related neurodegeneration. Serum miRNAs are a possible non-invasive marker for AD diagnosis, and may also be useful in differentiating MCI and AD [104]. MiR-206 and miR-132 was upregulated in MCI, and their serum levels also correlated to the degree of cognitive decline. These miRNAs can regulate the expression of different genes (such as BDNF or SIRT1), and may be associated with learning and memory. In addition, circulating miR-206 can predict the MCI conversion to AD [101]. MiR-613 has also been predicted to downregulate BDNF expression, and its levels were elevated in both serum and CSF of AD and MCI patients. MiR-613 levels of were higher in the AD group, compared with the individuals with MCI. Thus, miR-613 may be a possible marker for predicting MCI conversion to AD [105]. Yang et al. (2018) screened miR-135a, miR-193b and miR-384 in serum exosomes, and found that they can be used as potential non-invasive markers for early AD diagnosis. Elevated miR-135a and miR-384 levels were reported in patients with AD and MCI, while the levels of miR-193b were decreased in patients. A combination of these three miRNAs can have a predictive value to estimate the risk of MCI onset and conversion to AD [106]. Reduced miR-107 levels were found in the plasma of patients with early MCI disease stages, compared with the unaffected individuals. This reduction was correlated with the severity of disorder, and may be effective in distinguishing between the individuals with AD and MCI. MiR-107 has been suggested to protect against disease progression and to accelerating the expression of beta secretase (*BACE1*) gene [107].

MiRNA expression can be screened by different methods, such as qualitative PCR (qPCR), microarray or next generation sequencing (NGS) techniques [108]. Microarray technologies are based on probe-target hybridization and fluorescence signal detection. They can measure the relative abundance of miRNAs from various samples. A microarray may be more cost-effective than miRNA sequencing, and can still analyze a large number of samples or miRNAs simultaneously. However, there may be a high risk of technical errors in miRNA experiments, and it could be difficult to design and conduct microarray associated experiments [109]. The qPCR methods could be useful for miRNA quantification and profiling. It could be used for the verification of array experiments or the analysis of a small amount of the target. Challenges can occur during qPCR, such as the short length of miRNA, the variable GC content, and high similarity between sequences of miRNAs belonging to the same family. Another limitation with qPCR and microarray is that these methods are unable to screen novel miRNA candidates [110]. MiRNA sequencing methods provide more accurate profiling of miRNAs. High throughput NGS could provide effective miRNA profiling. Several NGS platforms are available, such as IonTorrent, Illumina or Solid. The recent NGS techniques could provide high resolution and high throughput of miRNA analysis. In addition, de novo miRNA discovery is also possible by sequencing techniques [108].

Multiple studies have addressed alterations in the miRNA profiles from the blood of MCI patients. So far, at least ten studies reported miRNAs that were identified as significantly different between MCI/AD and healthy subjects. Table 4 summarizes the results of a review of these recently published studies. Nagaraj et al. (2017) suggested a set of six microRNAs in blood plasma that distinguished MCI from normal control (NC), and which comprised four upregulated mRNAs (483-5p, 486-5p, 200a-3p, 502-3p) and two downregulated mRNAs (30b-5p, 142-3p) [111]. Liu et al. (2018) showed that let-7b expression in CD4+ lymphocytes isolated from CSF cell pellets of AD and MCI individuals was significantly higher than those of subjective memory complaints (SMC) patients [112]. In a large group consisting of 57 AD, 37 MCI, and 40 NC individuals, no differences in microRNAs levels were found using qPCR between AD, MCI, and NC after correcting for confounding factors including age, gender, sample storage time, and centrifugation status [113]. In the frontal cortex, miR-498 and miR-150 were found to be significantly upregulated in AD, and miR-150 was found to be upregulated in MCI, compared with NC [114]. In addition, two distinct clusters, miR-212/miR-132 and miR-23a/miR-23b, were significantly downregulated in MCI [114]. It was recently reported that several upregulated microRNAs (483-5p, 486-5p, 200a-3p, 502-3p) and 2 downregulated microRNAs (30b-5p, 142-3p) could be used to distinguish MCI from NC [111]. Kayano et al. (2016) used RT-qPCR screening of microRNAs in blood plasma of 23 MCI and 30 NC subjects to identify 20 pairs of microRNAs that can distinguish MCI from NC [115]. Nagaraj et al. (2017) performed RT-qPCR validation with blood plasma from 20 AD, 15 MCI and 15 NC and found that increased levels of miR-486-5p and miR-483-5p were the most significant indicators of MCI and AD [111]. In addition, upregulation of miR-502-3p and miR-200a-3p was observed of MCI and AD. Further, upregulation of miR-502-3p and miR-200a-3p was observed in MCI and AD relative to NC.

As mentioned earlier, most studies were focused on the analysis of only those miRNAs, whose deregulated expression had previously been previously identified to be linked to pathological development of the disease. This approach provides an opportunity to study the underlying mechanisms involved in MCI/AD etiology, which might not have been previous associated with the disease. With the advantage of the potential biomarkers’ known connection to disease withstanding, there are a couple of drawbacks to this biomarker. Due to the nature of circulating fluids being the common reservoir for all secreted molecules from all organs and tissues, the candidate miRNA biomarker may be involved in diseases of various other organs, hence making the correlation harder. In addition, despite of rapidly growing number of studies on diagnostic applications of miRNA, further studies are needed to verify their role in diagnosis of MCI. One factor impeding the progress in the field is the difficulty of comparing the data reported by different groups due to the use of different methods for searching for potential circulating miRNA biomarkers as well as different techniques for miRNA measurement and data normalization. The detection of such common processes is useful for the monitoring of normal brain aging and the diagnosis of MCI, which is a syndrome characteristic of an early stage of the disease. However, such a test will not predict MCI outcome. This goal could be accomplished by other tests, such as CSF protein analysis or imaging techniques, or by different miRNA biomarkers specific for various AD stages. However, the use of miRNAs biomarker for disease detection was still challenged in some cases.

## 8. Genetic factors, Which Could Increase the Risk for MCI-AD Conversion

Several genes have been suggested to cause AD [116], affect the risk of disease onset, or act as disease modifying factors [117,118,119,120]. Genetic markers may be useful in predicting the conversion of MCI into AD or dementia [56,121,122]. The genetics of MCI remains unclear, however, cognitive abilities and cognitive decline could be heritable [31,117,118,123,124,125,126,127,128,129].

The apolipoprotein (*APOE*) ε4 allele was verified as the strongest risk factor for late onset AD [130,131], and it may impact the onset of MCI. The ε4 allele can increase the risk of MCI, even one ε4 allele can increase the risk of amnestic MCI six times compared with ε3/ε3 carriers [130]. Contradictory studies are present regarding whether the A*POE* ε4 allele increases the risk of conversion of MCI to dementia [132]. *APOE* ε4 status can lead to reduce Aβ and increased Tau in CSF in MCI patients and unaffected controls. Espinosa et al. (2018) found a significant association between different memory functions (delayed recall, learning and recognition memory) and the *APOE* ε4 allele in MCI patients [133]. These findings suggest that the *APOE* ε4 allele may increase the risk of MCI conversion into AD [130]. Furthermore, besides APOE, several strong AD risk genes may also increase the risk of MCI. No direct association was found between SORL1 and MCI. However, SORL1 expression was reduced in the brain of MCI patients, and may affect disease severity [59,116,119,121]. LRP6 is a co-receptor in WNT signaling and plays an important role in brain functions by maintaining synaptic structure and function. A deficiency in the *LRP6* gene could cause memory impairment by affecting learning and memory. LRP6 may be involved in the onset of neurodegeneration through dysfunctions of long-term potentiation and immune activation. Abnormal LRP6 could also result in amyloid production and aggregation [134,135]. Espinosa et al. (2018) also examined several additional genes among MCI patients and screened for their cognitive functions. Polymorphisms in *TOMM40* were described previously as a risk factor for MCI–AD progression. Some variants may also be associated with reduced performance on the delayed recall test. TOMM40 may also affect age-related memory functions [133]. Mouse models have revealed that TLR4 can affect early stages of neurodegeneration, and MCI through the impairment of microglia activation. Normally, TLR4 signaling plays a role in the clearance of amyloid peptides and protects nerve cells against neurodegeneration [109]. Genetic variants in *CLU* can also affect cognitive functions by altering amyloid-and lipid (cholesterol) metabolism [56]. *CLU* haplotypes (such as the combination of rs1532278, rs9331888 and rs11136000) could affect cognitive performance, and may be associated with memory impairment [136]. Variants in the *CLU* gene could affect the clusterin levels in plasma and possibly predict MCI progression into AD [56,137]. Estrogen receptor (ER) genes (ESR1 and ESR2) can be expressed in the brain (hippocampus and amygdala), but their role in neurodegeneration remains unclear. Different alleles in ESRs have been examined (rs9340799, rs2234693, and rs2228480 in the ESR1 gene and rs4986938 in the ESR2), but these variants may not be independently associated with AD or MCI. However, combining these variants with APOE ε4 alleles can increase the risk for both amnestic MCI and AD, especially in women [138]. Angiotensin converting esterase (ACE) can impact amnestic MCI. A common insertion/deletion polymorphism in intron 16 could affect the white matter integrity inside the brain. A greater number of homozygous D-allele (deletion) was observed in MCI patients than in controls. It may also be associated with increased serum ACE levels and lower fractional anisotropy (FA) values in different brain areas (such as the middle frontal gyrus and the left anterior cingulate). The D-allele in ACE and elevated ACE levels in serum may serve as potential risk factor for MCI [139].

Among other biomarker candidates, the use of genomic technologies is valuable in identifying potential biomarkers in several neurological diseases, including MCI and promises to provide important insight for the future in terms of personalized diagnosis. There are two main strategies used to identify the genetic risk factors of MCI. The first strategy employs a phenotype to genotype approach. Researchers examine polymorphic genomic markers such as short tandem repeats found commonly in families with a high burden of MCI-AD across multiple generations to identify broad genomic regions co-transmitted with the disease. The second strategy for identifying the genetic risks of AD employs a genotype to phenotype approach. These studies are known as genome-wide association studies and represent modern genetic tools for studying complex or sporadic forms of MCI and AD [140], providing the biological basis of the disease. However, genetic testing can provide only limited information about an inherited condition. The test often cannot determine if a person will show symptoms of a disorder, how severe the symptoms will be, or whether the disorder will progress over time. Another major limitation is the lack of treatment strategies for many genetic disorders once they are diagnosed.

## 9. Challenges in MCI Diagnosis

Even though several biomarkers for MCI have been discovered in the blood, it may be difficult to predict disease occurrence during the preclinical stage. It may be difficult to distinguish healthy controls from AD progression when using plasma markers [31]. In addition, differentiating individuals with MCI that progress to dementia from individuals who do not develop dementia has yet to be archived [141].

MCI presents in diverse ways with several distinguishable subtypes, such as amnestic MCI, executive MCI. However, categories of the disease may not be uniform, based on the findings of different studies. The majority of publications categorized MCI into four subtypes. Petersen categorized the MCI into single- and multi-domain amnestic and non-amnestic MCI [142]. Rosenberg et al. identified four types of disease status, including amnestic or executive MCI, both amnestic and executive MCI, or neither. Their categories were based on several parameters, including language, memory or visuospatial functions [143]. Mansbach et al. introduced similar categories based on verbal memory, executive functions and attention capacity [144]. Putcha and Tremont (2016) [145] and Albert et al. (2011) [146] divided MCI into amnestic and non-amnestic MCI based on executive function, attention, and episodic memory. Several additional factors could affect the categorization of MCI, such as education, culture and social interactions [147]. Improving the accurate diagnostic criteria is important because not every MCI-affected individual will develop AD or other dementia, and many MCI-affected individuals can be stabilized or may even revert. MCI should not be overlooked, and it is important to reduce false positive diagnosis, and to differentiate individuals whose cognitive function could continue to decline from those who will remain stable [148].

The other issue with MCI diagnosis is that no specific biomarkers are available. The majority of CSF biomarkers are based on individuals who were diagnosed with MCI, and there may be a high risk for misdiagnosis. There may be pathological overlap between different diseases, such as AD, dementia with Lewy Bodies (DLB), or vascular dementia [116,121,127]. It is also unclear what type of disease would occur in patients in the future. Similarly, plasma and serum biomarkers may not present accurate disease diagnoses or reflect exact progression.

## 10. Discussion and Future Perspectives

Research into the possible biomarkers capable of detecting etiological factors and predicting the progression of MCI is constantly growing. Identifying the subjects at a higher risk of progressing from MCI to AD is essential for effectively managing this condition. The ideal markers should be able to distinguish the different subtypes of MCI (amnestic-and non-amnestic), which may also improve the prediction of risk for the AD and other kinds of dementia onset [149]. A biological marker, or biomarkers, is defined as “a characteristic that is objectively measured and evaluated as an indicator of normal biological processes, pathogenic processes, or pharmacological responses to a therapeutic intervention” [30,101,150,151,152]. Analysis of biomarkers from blood, urine, and cerebrospinal fluid, combined with imaging data, could help to predict the prognosis of diseases. The A/T/N marker system has been suggested as a useful tool for MCI analysis, and improved its diagnosis and prognosis. However, several limitations have been observed when using this system in MCI patients, and these issues should be addressed in future studies. One such problem with the A/T/N system is that it may be limited to the amnestic form of MCI, and it may be difficult to analyze its non-amnestic forms and other atypical cognitive dysfunctions. Further validation of A/T/N classifications is needed in a large population. Longer follow-up may also be needed for MCI patients who have the potential to progress to AD [27].

There is a great interest of identifying novel biomarker candidate for cognitive dysfunctions. In clinical routine, we currently use the CSF biomarkers tau and Aβ1–42 for the differential diagnosis of MCI and AD. The issue with biomarker candidates is that they are often identified from small populations. These candidates should be studied and validated in a large number of individuals. Markers and marker candidates should be studied not only as research tools, but also in clinical settings [54]. Ideally, a biomarker should be able to detect a fundamental pathological feature of a disease; it should be validated in pathological proven cohorts, and should be precise, reliable, economical, and detectable by means of a non-invasive and simple procedure.

## Figures and Tables

**Table 1:** A/T/N markers and patterns of brain atrophy in mild cognitive impairment, compared with normal controls and AD patients. Individuals, who were positive for A, T and N markers may have elevated risk for both cognitive decline and MCI to AD progression.

	Controls	MCI Patients, Remained Stable	MCI Patients, Progressed to AD	AD
**A+T+N+**	19%	29%	54%	63%
**A+T+N−**	9%	19%	30%	19%
**A+T−N−**	18%	11%	5%	10%
**A−T+N−**	10%	6%	1.5%	2%
**A−T+N+**	7%	5%	1.5%	2%
**A−T−N−**	2%	NA	NA	NA
**A+T−N+**	NA	NA	NA	NA
**A−T−N−**	43%	31%	8%	4%

**Table 2:** Examples of studies that evaluated CSF β_1–42_ (Aβ_1–42_), total tau (t-tau), phosphorylated tau (p-tau) as potential biomarkers for MCI or AD-MCI.

Diagnosis	Aβ1–42 (pg/mL)	t-tau (pg/mL)	p-tau (pg/mL)	Diagnostic Criteria	Findings	Reference
Controls (*n* = 28)	721	177	34	MMSE & MDB	Aβ1-42 and p-tau predictive in MCI-AD conversion	Parnetti et al. (2012) [43]
MCI (*n* = 58)	919	261	41			
MCI-AD (*n* = 32)	480	475	90			
AD (*n* = 28)	446	680	72			
Cut offs	1372	416	59			
Controls (*n* = 114)	205.63	69.65	24.84	NINCDS-ADRDA	Tau and Aβ42 abnormalities are cognitive decline marker	Okonkwo et al. (2010) [44]
MCI (*n* = 95)	163.31	103.54	35.68			
AD (*n* = 100)	143.51	121.57	41.73			
Cut offs	192	93	23			
Controls (*n* = 94)	1325	217	19.0	NIA-AA	Tau/Aβ ratios may be accurate marker for MCI/AD	Hansson et al. (2018) [45]
Early MCI (*n* = 272)	1066	234	20.7			
Late MCI (*n* = 152)	784	291	28.0			
AD (*n* = 128)	595	340	33.8			
Cut offs	880	0.33	0.028			
Controls (*n* = 41)	503.99	86.03	41.59	NINCDS-ADRDA	CSF biomarkers could have successful predictive value of AD/dementia	Forlenza et al. (2015) [46]
MCI (*n* = 68)	410.91	88.38	45.92			
AD (*n* = 41)	328.76	145.69	66.72			
Cut offs	416	76.7	36.1			

MMSE & MDB = Mini Mental State Examination and Mental Deterioration Battery; NINCDS-ADRDA = National Institute of Neurological and Communicative Diseases and Stroke/Alzheimer’s Disease and Related Disorders Association; NIA-AA = National Institute on Aging and Alzheimer’s Association; AD-MCI = Alzheimer’s disease—Mild Cognitive Impairment; CSF = cerebrospinal fluid.

**Table 3:** MCI diagnosis approaches and their advantages/disadvantages.

Tool	Basic Properties	Advantages	Disadvantages	Reference
MDS	ELISA assay, which measures the toxic soluble Aβ oligomers in blood	Easy to perform, accessible, non-invasive, cost-effective, compared with CSF methods	Lower sensitivity than CSF methods. Level of blood biomarkers may be lower in plasma, compared with CSF	[30,31,32,33]
Simoa	Magnetic bead immunoassay on microfluidic array, detects oligomers in any biological fluids	Sensitive, quick, precise, flexible method, requires small sample size,	Requires special tool, higher cost	[35,36,37]
SQUID	Detects interactions between magnetic nanoparticles and biomarkers in any biological fluids	High sensitivity, flexible method, several markers can be monitored	Requires low temperatures, higher cost	[38,39]
CSF markers	Imaging and immunoassay methods, which screen Aβ42/Aβ40 ratio and Tau. Additional candidates were also discovered	Sensitive method, useful in differential diagnosis, useful in early diagnosis of cognitive decline	Higher cost, requires higher sample size, difficult to obtain	[48,49,50,51]

**Table 4:** MicroRNAs in human patients with AD or mild cognitive impairment.

Reference	No. of Patients, Gender	Mean Age/Mean MMSE	Source	Screening Method/Validation Method	Dysregulated miRNAs	Functional Outcomes, Specificity and Sensitivity
Nagaraj et al. (2017) [111]	7M/8F	68.1 years/score 25.9	Plasma without hemolysis and blood cells	RT-qPCR	Increased levels of miR486 and miR483-5p were the most significant indicators of MCI and AD. Also, upregulation of miR502-3p and miR-200a-3p in MCI and AD compared with NC was observed.	ROC indicated that miR483-5p and miR-502-3p are good tests to distinguish AD from NC, and MCI from NC (AUC > 0.9, specificity and sensitivity > 0.8, repeatedly, in both screening and validation studies).
Müller et al. (2016) [113]	15M/22F	73.1 years/score 24.8	CSF	qPCR	Increased expression levels of miRNA-146a in MCI compared with NC were lost when confounding factors were considered. Similarly, increased expression levels of miRNA-27a, -125b, -146a in MCI compared with AD were lost after correcting for confounding factors.	After correcting for confounding factors, no differences in miRNA levels were found between AD, MCI and NC
Weinberg et al., (2015) [114]	5M/5F	82.9 years/score 28.0	Frontal and interior temporal cortex obtained at postmortem (60% MCI as Braak stages III-VI)	Microarray/qPCR	miR-150 was upregulated in MCI, compared with NC. Also, two distinct clusters miR-212/miR-132 and miR-23a/miR-23b were significantly downregulated in MCI	SIFT1 mRNA levels were significantly upregulated by 40% in frontal cortex of MCI compared with AD and NC
Liu et al., (2018) [112]	19M/17F,	72.4 years/score 56.6	CSF	RT-PCR	Let-7b was significantly increased in MCI compared with SMC. Let-7b expression in CD4^+^ lymphocyte population from MCI was higher than SMC.	Addition of let-7b improves diagnostic performance of Aβ40 and Aβ42, and of t-tau and p-tau
Kayano et al., (2016) [115]	11M/12F,	72.8 years/score 24.3	Plasma	RT-qPCR	Differential correlation analysis was applied to the data set with 85 miRNAs. The 20 pairs of miRNAs which had the difference of correction coefficients > 0.8 were selected as biomarkers that distinguish MCI from NC	Two miRNA pairs miR-191/miR-101 and miR-103/miR-222 have the highest value AUC 0.96 and are good tests to distinguish MCI from NC. Also, miR-191 and miR-125b and miRN-590-5p have a high AUC > 0.95

AD = Alzheimer’s disease; MCI = mild cognitive impairment; NC = Normal control; M = Male; FT = Female; RT-qPCR = Quantitative reverse transcription PCR; CSF = Cerebrospinal fluid; MMSE = Mini-Mental State Examination.
